# Strong and Bitter Vegetables from Traditional Cultivars and Cropping Methods Improve the Health Status of Type 2 Diabetics: A Randomized Control Trial

**DOI:** 10.3390/nu13061813

**Published:** 2021-05-26

**Authors:** Anne Cathrine Thorup, Hanne Lakkenborg Kristensen, Ulla Kidmose, Max Norman Tandrup Lambert, Lars Porskjær Christensen, Xavier Fretté, Morten Rahr Clausen, Steen Møller Hansen, Per Bendix Jeppesen

**Affiliations:** 1 Department of Clinical Medicine, Aarhus University Hospital, Palle Juul-Jensens Boulevard 165, 8200 Aarhus N, Denmark; acth@seges.dk (A.C.T.); mntl@clin.au.dk (M.N.T.L.); 2 Department of Food Science, Faculty of Technical Sciences, Aarhus University, Agro Food Park 48, 8200 Aarhus N, Denmark; hanne.kristensen@food.au.dk (H.L.K.); ulla.kidmose@food.au.dk (U.K.); mortenr.clausen@food.au.dk (M.R.C.); 3 Department of Green Technology, Faculty of Engineering, University of Southern Denmark, Campusvej 55, 5230 Odense M, Denmark; lpc@igt.sdu.dk (L.P.C.); xafr@kbm.sdu.dk (X.F.); 4 Center for Clinical Research, Vendsyssel Hospital, Aalborg University, Bispensgade 37, 9800 Hjoerring, Denmark; steenmllerh@gmail.com

**Keywords:** type 2 diabetes mellitus, vegetables, cultivars, glucose tolerance, phytochemicals and sensory analysis

## Abstract

Vegetables rich in bitter-tasting phytochemicals may exert enhanced beneficial effects against key factors associated with type two diabetes (T2D). This study investigates whether selected cultivars of bitter and strong-tasting (BST) *Brassica* and root vegetables exert greater health benefits on T2D patients compared to equivalent modern mild and sweet tasting (MST) vegetables. A 12-week randomized, controlled, parallel intervention study involved 92 T2D patients, who were allocated three different diets: (1) 500 g daily of bitter and strong-tasting (BST) vegetables; (2) 500 g daily of mild and sweet-tasting (MST) vegetables; (3) 120 g daily MST normal diet (control). Both vegetable diets contained root vegetables and cabbages selected based on sensory differences and content of phytochemicals. Prior to and after the study, all participants underwent an oral glucose tolerance test (OGTT), 24 h blood pressure measurements, DEXA scans, and fasted blood samples. Both diets high in vegetables significantly reduced the participants’ BMI, total body fat mass, and HbA1c levels compared to control, but in the BST group, significant differences were also found regarding incremental area under the curve glucose 240 min (OGTT) and fasting glucose levels. A high daily intake of root vegetables and cabbages showed significant health improvements in both vegetable groups. BST vegetables had the greatest impact on insulin sensitivity, body fat mass, and blood pressure compared to control; moreover, they further improved glycemic control compared to MST vegetables.

## 1. Introduction

Type 2 diabetes mellitus (T2DM) is emerging as one of the greatest global health challenges of the 21st century. Over the last few decades, there has been an alarming increase in the prevalence of T2DM worldwide; in 2015, 415 million people had diabetes, and this number is expected to increase by 50% in 2040 [1]. T2DM develops due to a complex interaction between genetic predisposition and lifestyle. The actual manifestation of the disease is preceded by a phase of impaired glucose regulation, in which cardiovascular risk is already increased. Particularly important lifestyle factors that promote and/or accelerate the progression of T2DM are “bad” nutritional habits coupled with a lack of physical activity [2]. Diet has long been linked to the development of T2DM, obesity, and cardiovascular disease (CVD). Dietary modification is one of the cornerstones in chronic disease prevention and management. Several benefits from a fiber-rich diet comprising a high intake of fruits and vegetables (F&V) on T2DM are well documented [3,4]. High F&V consumption has been associated with decreased incidence and mortality from a variety of obesity-related diseases including T2DM and CVD [4,5]. In particular, an increased intake of green leafy vegetables is shown to be protective against the development of T2DM [6]. 

Vegetables provide a significant part of human nutrition, and they are important sources of nutrients, dietary fiber, and phytochemicals. Root vegetables and cabbages constitute a part of a habitual diet in many European countries [7]; they are important ingredients in the New Nordic Diet [8]. The Nordic diet comprises high intakes of traditional and locally sourced foods from Scandinavian countries. It is characterized by high intake of fruits and vegetables (particularly berries, kale, and root vegetables), legumes, potatoes, fresh herbs and mushrooms gathered from the wild, nuts, whole grains, dairy (low-fat), meat from livestock and game, fish, shellfish, and seaweed [8]. It is well accepted by nutritionists and scientists that consumption of root vegetables and cabbages can reduce incidence of chronic diseases such as T2DM, CVD, and cancer [9,10,11,12]. These health benefits are not only attributed to the dietary fiber content but also to the diverse phytochemical content of root vegetables and cabbages. Phytochemicals have direct and indirect beneficial effects on human health.

Phytochemicals exert these benefits through various physiologic mechanisms, exerting antioxidant activity, imitating hormones and modulating the immune system [10,13,14,15]. Phytochemicals also contribute to the color, taste, and smell of the vegetables [16]. Root crops (mainly Apiaceae family) and cabbages (Brassicaceae family) are coarse vegetables and rich in vitamins, dietary fibers and phytochemicals such as glucosinulates, polyphenols, phenolic acids, anthocyanins, polyacetylenes, and flavones (among others) [17,18,19]. Glucosinolates, besides having a potential beneficial effect on human health, are also responsible for the bitter acidic taste of Brassica vegetables, and the degradation products of glucosinolates, such as isothiocynates, nitrils, and thiocyanates, are responsible for the hot and pungent taste of mustard that is usually associated with the consumption of these vegetables [16]. In Apiaceae root vegetables such as carrots, bitter taste has been shown to be strongly correlated with their content of polyacetylenes and caffeic acid derivatives [20]. 

Many modern consumers dislike the strong and bitter tasting vegetables and prefer mild and sweet-tasting vegetables [21]. This has led to the selective cultivation of sweeter, more productive, and visually attractive cultivars of vegetables at the expense of nutrient and especially phytochemical content. Traditional cultivars may represent a larger diversity in taste and content of sensory and health beneficial compounds. T2DM can be reversed with diet; brassicas and apiacaeas are rich in phytochemicals and low in calories, different cultivars are different in taste and may have different metabolic effects, and the present article investigates this. We hypothesize that bitter and strong vegetable cultivars (mostly traditional cultivars) have a higher beneficial impact on human risk factors related to T2DM than mild and sweet-tasting modern vegetables and control diet (habitual diet consumed in Nordic countries). This is the first study to investigate whether high intake of selected cultivars of traditional (gene bank derived) bitter-tasting vegetables exert beneficial effects against T2D compared to habitual Nordic diet. 

## 2. Materials and Methods 

### 2.1. Study Design 

The study was carried out as a 3-month randomized, controlled, parallel-designed trial. The primary endpoint was change in insulin sensitivity assessed by area under the curve (AUC) of glucose 240 min from the oral glucose tolerance test (OGTT). Secondary endpoints were change in glucose, HbA1c, insulin, glucagon, insulin resistance, lipids, body composition, and blood pressure. 

**Figure 1:**
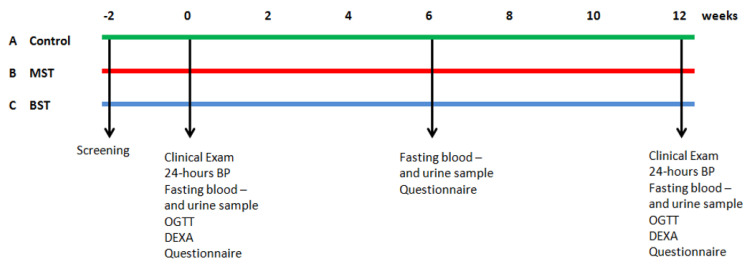
The study design. The far left shows the three groups in the top (**A**) the control group (green); (**B**) mild and sweet tasting (red); and finally (**C**) bitter and strong tasting (blue). Screening took place approximately 2 weeks prior to project start. At weeks 0 and 12, fasting blood and urine samples were taken, 24 h blood pressure (BP) and oral glucose tolerance test (OGTT) were performed, and DEXA scans were conducted. At weeks 0, 6, and 12, participants filled out thorough questionnaires. Randomization took place at week 0.

The study includes three different groups provided with different diets (Figure 1):

Group A: A diet habitually consumed in the Nordic countries, 120 g daily MST root vegetables and cabbages (normal control diet).

Group B: Daily intake of 500 g Mild and Sweet-Tasting (MST) root vegetables and cabbages.

Group C: Daily intake of 500 g Bitter and Strong-Tasting (BST) root vegetables and cabbages.

The root vegetables and cabbages were handed out once a week during a verbal meeting to ensure that the participants followed their respective groups’ diet. Groups B and C received 7 kg of root vegetables and cabbages weekly in pre-packed boxes, ensuring an adequate quantity for each week. Groups B and C also received easy recipes for inspiration to help them prepare, cook, and consume the high daily amounts of root vegetables and cabbages. They were also provided with written dietary advices as well as guidance on cooking and storage of the root vegetables and cabbages. The participants were instructed to weigh the prepared vegetables prior to cooking. The recipes were composed by a leading Danish chef Per Mandrup and a dietician Susanne Elman Pedersen from the Danish Diabetes Association. The control group (A) continued on their normal diets but were required to meet up each week to collect a box of normal groceries and to ensure their participation in the study. At the end of the study, participants in group A were offered weekly training by a physiotherapist and counseling with a registered dietician for 12 weeks as an acknowledgement for their participation in the study. 

### 2.2. Ethics Statement 

The study was conducted according to the guidelines in the Declaration of Helsinki and approved by The Danish Ethical Committee (M-20110068), The Danish Data Protection Agency, and the study protocol was registered at ClinicalTrials.gov (NCT01397942). Participants provided written informed consent prior to enrollment into the trial.

### 2.3. Participant Recruitment and Screening

Participants were recruited from the general population from 2011 to 2013 in the Northern part of Denmark via press release, by advertisement within the local paper, and by local medical clinics. Interested people were screened for eligibility by telephone at first, and if they met the inclusion criteria, they were invited into the clinic for a screening visit. The screening visit consisted of an extended interview followed by a fasted blood sample. Basic anthropometric measurements were also recorded. The inclusion criteria were as follows: T2DM in diet treatment or on oral anti-diabetic drug; age 30–70 years, both sexes; HbA1c > 36.6 mmol/mol and <56.3 mmol/mol; BMI 23–40 kg/m^2^. The exclusion criteria were as follows: in treatment with systemic glucocorticoids, insulin, victoza, GLP-1 analogous, or glitazones due to fluctuation in the blood glucose; participation in other clinical trials; cardiovascular, chronic liver, thyroid, or kidney diseases; a disease or condition, which may influence the participants’ ability to follow the dietary intervention protocol; alcohol or drug abuse; pregnancy or breast feeding; fasting plasma glucose <4 mmol/L or >12 mmol/L and finally blood pressure >160/110 mmHg. Eligibility or exclusion was assessed by the research team based on the screening visit.

**Figure 2:**
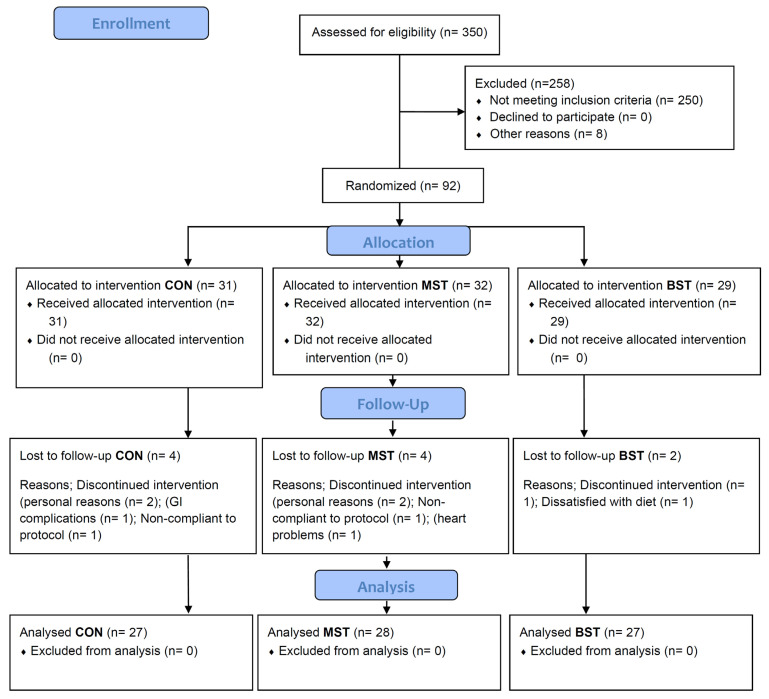
Flow chart of the participants in the study with the BST, MST, and control diet. BST; bitter strong tasting. MST; mild sweet tasting.

Approximately 350 enquiries from the general public led to 105 people undergoing the screening visit, but only 92 people met the inclusion criteria and were enrolled in the study (Figure 2). Three participants dropped out due to personal reasons; one was excluded by the research team, and one dropped out due to dissatisfaction with the diet. A total of 82 successfully completed the trial.

Participants were randomized at study entry but were not informed about their randomization allocation; however, they were given different guidelines. A computer-generated randomization list was used to allocate the participants into one of the three groups: A, B, and C. The research team was aware of which group the participants were allocated to. The study was open label to the research team but blinded to the participants, as the boxes were identical and masked so the contents were only visible when opened at home.

The participants were instructed to keep physical activity, smoking, medicine use, and drinking habits constant during the study period and were told to maintain their normal diet apart from the vegetables given. The use of anti-diabetic, anti-hypertensives, and lipid-lowering drugs should be continued during the intervention without changing the dose; however, due to high levels of vitamin K in cabbages, participants on vitamin K antagonist drug Marevan (Warfarin) were monitored extra. Only sulfonylurea and metformin anti-diabetic drugs were allowed during the trial. Treatment with systemic glucocorticoids, insulin, Victoza, GLP analogues, or glitazones was excluded. These parameters were monitored throughout the study, and participants were informed to alert the research team if they were considering changing medication.

### 2.4. Study Assessments

The study was carried out at Center for Clinical Research, Vendsyssel Hospital, Aalborg University. Study researchers met the participants once a week at the hand-out to discuss their study orders, monitor compliance, and discuss any individual difficulties that might have occurred. At week 0 and 12, a thorough questionnaire on dietary intake, exercise habits, alcohol and tobacco use, and lifestyle choices was used to ensure no alterations in the participant’s lifestyles during the intervention period. Only the dietary intake in groups B and C were allowed to be altered due to the high amount of daily intake of root vegetables and cabbages. 

### 2.5. Vegetables 

The BST and MST diets included curly kale, white cabbage, pointed cabbage, red cabbage, celeriac, carrot, and beetroots. Vegetables were field-grown at Aarhus University (Aarslev, Denmark) under the agronomic conditions given in Groenbaek et al. (2014) [22]. A small number of carrots and beetroots were grown by commercial growers Lammefjorden (Højby, Denmark) and Tange Gartneri A/S (Bjerringbro, Denmark). The seeds of the traditional cultivars were provided from the NordGen germplasm collection (NordGen, Alnarp, Sweden) and of the modern cultivars from SeedCom A/S (Vissenbjerg, Denmark). The cultivars, storage, and cropping methods to compose the BST and MST diets were selected based on an initial screening by a trained sensory panel resulting in the BST and MST diets described in Table 1. The sensory selection was supported by partly testing the content of phytochemicals; however, this is not included in this paper, as this is beyond the scope of the article, as there are hundreds of compounds for each vegetable. The vegetables were harvested, cleaned, and put in coded boxes 1–5 days prior to delivery at the study clinic at Hospital Vendsyssel. Storage and transport were under cool and humid conditions to ensure high quality at delivery.

In order to determine whether the BST vegetables were in fact more bitter and strong tasting and the MST vegetables were sweeter and milder, a simple sensory discrimination test was carried out for the same type of vegetables. Random selected samples of vegetables were analyzed by a trained independent sensory panel during the 12-week intervention period. The taste of the vegetables in the BST and MST boxes was validated by a trained sensory panel of 9–11 assessors using a sensory triangle test to validate if the taste differs among the vegetables between the two boxes. The assessors in the sensory panel were tested and trained in accordance to international standards ISO 8586-1 (ISO, 1993) [23]. The sensory evaluation was carried out in a sensory evaluation laboratory fulfilling the requirements provided by the ASTM international (ASTM, 1986). The test was carried out as a triangle test with two identical samples of BST and one sample that was different from the two samples. In order to avoid bias, the samples were coded and served randomly so that some of the assessors received two identical samples of BST vegetables and one sample of MST vegetable, whereas others received two samples of MST vegetable and one of BST vegetable. In addition, the assessors noted which one(s) of the samples were the sweetest, the most bitter, and the strongest tasting one(s). Sensory evaluation of kale was conducted on samples that were prepared from the leaves with only 3–4 cm stem. The outer leaves of white cabbage were removed before the heads were cut in quarters. Both 207 kale and white cabbage were chopped in small pieces. The outer part of celeriac was removed. The top and bottom of carrot were removed. Both carrot and celeriac were shredded in 2 mm thick sticks. The vegetable samples were served in small plastic beakers with lids (ABENA A/S Aabenraa, Denmark) in amounts of approximately 15 g. All the vegetable samples were served raw and in 3 replicates in order to obtain between 27 and 33 answers per vegetable type for each testing date. Red light was used in the booths during evaluation in order to mask differences in appearance. 

### 2.6. Baseline Characteristics 

Body composition: At week 0 and 12 standing height, weight (participants lightly clothed) and waist circumferences were measured. Body composition was measured using a whole-body dual-energy X-ray absorptiometry (DEXA) scan at weeks 0 and 12. All scans were performed by a trained radiographer using a Norland XR-800 dual-energy X-ray absorptiometry scanner (Norland Cooper, Surgical, Trumbull, Connecticut, USA). Blood pressure: 24 h blood pressure measurements were performed at weeks 0 and 12 using a SpaceLab monitor (Spacelabs Medical, Redmond, WA, USA). The participants would come into the clinic in the morning, and a trained study researcher applied the blood pressure monitor to the right upper arm. The monitor was pre-programmed to measure the blood pressure hourly [23], and the participant were given a thorough oral instruction not to move or talk during the measurements and relax their arms in an extended position during measurements. The participants were also given a form to write down any unusual activity during the 24 h of measurements as well as their individual bedtimes. Participants were advised to carry out their usual activities but avoid strenuous exercise. Daytime was defined as measurements from 6:00 a.m. to 11:00 p.m. and night-time was within the timeframe of 11:00 p.m. to 6:00 a.m.

Oral Glucose Tolerance Test: Participants underwent an OGTT in weeks 0 and 12. The participants came into the study clinic after an overnight fast and were given a glucose load of 75 g D-glucose just after the blood sample was taken to time 0. Blood samples were taken to the times: −30, −15, 0, 10, 20, 30, 45, 60, 120, and 240 min. Participants were instructed to take their usual medication on both OGTT days. Afterwards, the participants were offered lunch.

### 2.7. Biochemical Analysis 

A fasting blood sample was collected at weeks 0 and 12 using an evacuated tube system and transferred to EDTA or lithium heparin evacuated tubes, temporarily stored on ice, and centrifuged at 4 °C at 3500× *g* within 30 min. Then, plasma was separated into aliquots and stored at −80 °C. All samples from each subject were analyzed within one batch to reduce inter-batch variation after study completion. All laboratory analyses were blinded, and all analytic procedures were carried out in accordance with the manufacturer’s instructions.

Plasma insulin was measured with an enzyme-linked immunosorbent assay (K6219, Dako, Glostrup, Denmark), and plasma glucagon concentration was determined by a radioimmunoassay kit (Millipore, Billerica, MA, USA). Plasma glucose was measured using an enzymatic reference method (Roche Diagnostics GmbH, Mannheim, Germany) on the Cobas c111 system. The fasting plasma lipid profile (total cholesterol, HDL, LDL, and triglycerides) was assessed using enzymatic colorimetric assays on the Cobas c111 system (Roche Diagnostics GmbH, Mannheim, Germany). FFA analysis was also assessed by a colorimetric assay on the Cobas c111 system (Roche Diagnostics GmbH, Mannheim, Germany) but with a WAKO kit (HR Series NEFA-HR (2), WAKO, Neuss, Germany). Total vitamin D content (25-hydroxyvitamin D2 and D3) was determined by electrochemiluminescence binding assay on a Cobas 8000 system (Roche Diagnostics, Indianapolis, IN, USA). P-parathyroid (PTH) was analyzed by electrochemiluminescence immunoassay on the Cobas 8000 system (Roche Diagnostics, Indianapolis, IN, USA). HbA1c was analyzed using HPLC on an HLC-723 GHb G7 (Tosoh Europe N.V., Tessenderlo, Belgium). Based on fasting plasma glucose and insulin concentrations, the homeostatic model assessment (HOMA) assesses insulin resistance (IR) by the HOMA-IR [24]. 

### 2.8. NMR Spectroscopy

Plasma samples prepared from fasted blood at weeks 0 and 12 were prepared for NMR spectroscopy by mixing 400 µL plasma with 200 µL of D2O. All NMR measurements were performed on a Bruker Avance 600 NMR Spectrometer (Bruker BioSpin, Rheinstetten, Germany) with 1H operating frequency of 600.13 MHz and equipped with a 5 mm TXI inverse probe. The Carr–Purcell–Meiboom–Gill (CPMG; cpmgpr1d, Bruker pulse sequence) pulse sequence with water suppression was applied for the acquisition of 1H NMR spectra of plasma samples at 310 K. The total spin–spin relaxation delay was 100 ms, the spin-echo delay was 1 ms, and the relaxation delay between pulses was 3 s. The spectra were acquired by 64 scans, 32k data points, a spectral width of 17.34 ppm, and an acquisition time of 1.57 s. 

Spectra were imported to Chenomx NMR suite v 8.0 (Chenomx Inc., Edmonton, AB, Canada) and were automatically phased and manually baseline corrected. Referencing of the ppm scale was based on the format singlet at 8.5 ppm, and identification of metabolites was based on the build in database. After integration, concentrations were calculated relative to the blood glucose levels measured enzymatically.

### 2.9. Analysis of Plasma for β-Carotene (Compliance) 

Fifteen random samples from each group were used in the compliance study. A simple and rapid extraction procedure was applied in order to minimize the exposure of the samples to light, heat, and air. Frozen plasma samples were thawed at room temperature and homogenized before sampling. Then, 400 µL of acetone (VWR Prolabo, Herlev, Denmark) were added to 400 µL plasma and then shaken with a vortex. The proteins were precipitated by centrifugation (5 min at 4000 RPM). The supernatant was recovered and filtered through 0.20 µm syringe PTFE filters (Frisenette ApS, Knebel, Denmark) before LC-MS analysis. LC-MS analysis was performed on a LTQ XL (Linear Quadrupole 2D Ion Trap Mass Spectrometer, Thermo Scientific, San Jose, CA, USA) operated in atmospheric-pressure chemical ionization (APCI) positive mode and equipped with an Accela HPLC Pump and a PDA detector. Settings for the mass spectrometer were 50, 10, and 3 (arbitrary units) for sheath, auxillary, and sweep gas flow rates, respectively, vaporizer temperature 450 °C, discharge current 5 µA, capillary temperature 275 °C, capillary voltage −36 V, and tube lens −65 V. Spectra were recorded in selected ion monitoring (SIM) mode of ion m/z 538 +/− 0.5 [M + H]+. Separation was performed on a reverse phase Phenomenex C30 column (150 × 4.6 mm i.d., particle size 5 µm, Torrance, CA, USA) at 30 °C, and the elution was performed by the solvent gradient A = methanol, B = hexane:isopropanol (50:50); 0 to 1 min isocratic 0% B, 1 to 14 min linear gradient from 0% to 75% B, 14 to 15 min linear gradient from 75% to 99% B, from 15 to 30 min isocratic 99% B, from 30 to 31 min linear gradient from 99% to 0% B, from 31 to 46 min isocratic 0% B. The flow rate was 1 mL min^−1^. Sample injection volumes were 10 µL. Tuning of the mass spectrometer was optimized using β-carotene from a stock solution (Sigma-Aldrich A/S, Copenhagen, Denmark). Dilutions of the stock solution of β-carotene were made to obtain a calibration curve (R2 > 0.99) used for the quantification of β-carotene in the plasma samples. All solvents were of HPLC grade (VWR Prolabo, Herlev, Denmark).

### 2.10. Statistical Analysis 

Statistical analysis included all participants who completed the study and from which appropriate material was obtained. Data were analyzed using StataIC statistical software (version11.2; StataCorp LP, College Station, TX, USA), and graphs were created by GraphPad Prism version 4 (GraphPad Software Inc., San Diego, CA, USA). Normal distribution of the data was checked by visual inspection, QQ-plots, and histograms. Baseline comparisons were assessed by one-way ANOVA test. Absolute change from baseline was calculated after the 12-week study period. One-way analysis of variance (ANOVA) with post hoc multiple comparison Tukey test was used to test for overall group differences; Kruskal–Wallis test was used for data that did not follow normal distribution and denoted by (†). Any differences within the groups were assessed by Student’s paired *t*-test. Data are presented as mean ± SEM. In all cases, *p* < 0.05 was considered significant. The primary endpoint was changes in AUCglucose 240 min from the OGTT. AUCglucose 240 min was calculated by the trapezium rule. The statistical analysis of the sensory data was based on a binomial distribution that was used in order to evaluate if there were significant differences between the samples based on the number of correct answers. Sample size calculation: Power calculations were performed for the primary outcome; insulin sensitivity was measured as AUCglucose 240 min from the OGTT. At 80% power and 5% significance, the minimum number of participants required to allow detection of a difference of 10% difference in AUCglucose 240 min from start to end was estimated to be 23. An a priori power calculation was used to calculate the sample size required to determine the effects of BST compared to habitual (control) diet. Power for BST vs. MST was not calculated, as there are no prior data for BST. A minimum of 29 participants was recruited to each group to allow up to a 20% drop-out rate.

## 3. Results

Eighty-two participants completed the 12 weeks of intervention, meaning ten participants dropped out (Figure 2). The intervention was implemented as intended, and there were no adverse events associated with the intervention. Participants were well matched at the time of randomization (week 0) with no statistical differences in baseline values between the three groups. Table 2 shows the mean and range of baseline characteristics of the subjects. 

### 3.1. Diabetes Status 

**Figure 3:**
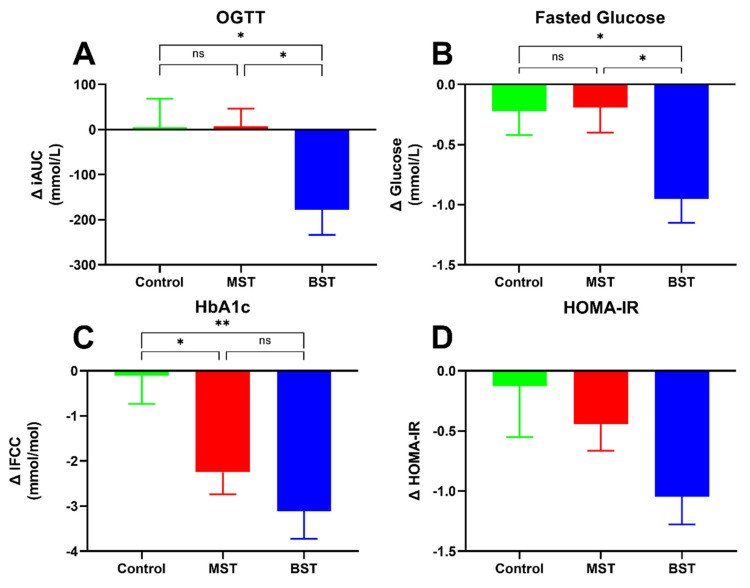
Change (Δ) in (**A**) area under the curve (AUC) glucose (240 min) during the OGTT, (**B**) fasting glucose changes, (**C**) HbA1c changes, and (**D**) changes in HOMA-IR; after 12-week intervention. *p* < 0.05 (*); *p* < 0.001 (**).

**Figure 4:**
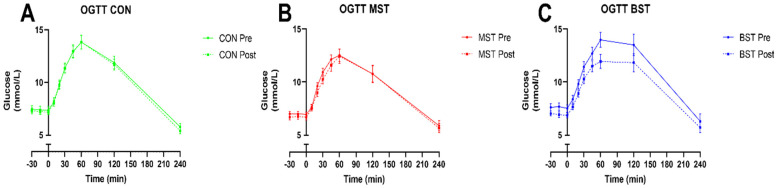
OGTT before and after 12-weeks of intervention. (**A**) CON, (**B**) MST, (**C**) BST. Data are presented as mean (±SEM). Significance testing was performed on between group differences in change in iAUC OGTT glucose (please see Figure 3A).

After the 12-week intervention period, changes in iAUCglucose 240 min (OGTT), fasting glucose levels, HbA1c, and HOMA-IR are shown in Figure 3. Regarding improvements in iAUCglucose 240 min (OGTT) (Figure 3A), we observed a significant reduction in iAUC for the BST group of −177.4 ± 55.62 mmol/L compared to CON and MST intervention (*p* < 0.05 respectively). The iAUC for the CON and MST group did not significantly change from baseline (Figure 4). The BST intervention also significantly lowered fasted glucose (−0.95 ± 0.20 mmol/L) compared to MST and CON groups (*p* < 0.05, respectively), while MST and CON groups remained unchanged from baseline (Figure 3B). In addition, HbA1c (Figure 3C) decreased in both the BST (−3.1 ± 0.61 mmol/L) group and in the MST of (2.25 ± 0.49 mmol/L) group compared to the control group (*p* < 0.01 and *p* < 0.05, respectively), which was unchanged from baseline. There was no significant difference between BST and MST groups in the reduction of HbA1c. Furthermore, there was no significant difference in change in HOMA-IR between the three groups (Figure 3D), although the intragroup change in HOMA-IR for the BST declined significantly (*p* < 0.0001, Table 3) The goal of maintaining the control group in their current health status was achieved, as no intra-group changes were seen after the intervention in any of the above-mentioned categories. Changes in fasting insulin and glucagon levels as well as AUC insulin 240 min and AUC glucagon 240 min from the OGTT are shown in Table 3. Fasting insulin levels did significantly decline within the BST (*p* < 0.05) and MST group (*p* < 0.01), but no changes were found between the groups. No significant changes were observed concerning glucagon levels, and no significant changes were observed in AUC insulin 240 and AUC glucagon 240 min from the OGTT. HOMA-IR did not change between the groups, but a significant intragroup decline was found in the BST group (*p* < 0.0001) and a tendency for reduction in the MST group (*p* = 0.055). There were no registered changes to participants’ medicine usage during the study. 

### 3.2. Body Composition 

**Figure 5:**
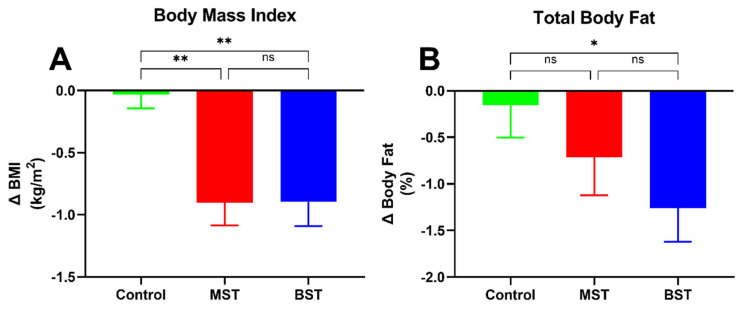
(**A**) Change (Δ) in BMI, (**B**) change in total body fat (†) percentage Data are presented as mean (±SEM). (*) *p* < 0.05, (**) *p* < 0.01.

We observed significant changes concerning the body composition in the BST and MST groups compared to control (Table 4). There were no significant intragroup changes in body composition in the control group. Both BST and MST groups had a significant decline in BMI (Figure 5) compared to control (*p* < 0.01 respectively) and weight (*p* < 0.01 and *p* < 0.001, respectively). No significant difference was found between the groups in waist circumference, and only the BST group had a significant reduction in total body fat mass compared to control (*p* < 0.01). 

### 3.3. Blood Pressure 

The results from the intervention regarding blood pressure are shown in Table 4. There were no significant intragroup decreases between 24 h systolic and diastolic blood pressure following 12-month intervention. Only the BST intervention showed a significantly reduction in change in diastolic blood pressure compared to control.

### 3.4. Lipids 

There were no significant differences in changes of lipids between any of the arms of the study (Table 5). Total cholesterol was significantly decreased within the BST group alone.

### 3.5. PTH and Vitamin D

The BST and MST groups experienced a significant decrease in the total vitamin D levels caused by the change in season. No changes were found regarding PTH levels (Table 5). 

### 3.6. NMR Spectroscopy 

Fasted plasma concentrations of selected metabolites were determined using NMR spectroscopy. Lactate concentrations significantly decreased intragroup in the BST group and MST group by −13.6 ± 5.3 % (*p* < 0.01) and −8.8 ± 5.7 % (*p* < 0.05), respectively. Only isoleucine decreased in the BST group −11.6 ± 4.5 % (*p* < 0.05). None of the metabolites changed in the control group. No changes were observed between the groups. 

### 3.7. Compliance Study 

The plasma level changes of β-carotene can be found in Table 5. The biggest increase was found in the BST group with a percentage increase of 9.44 ± 5.1%, although it was not significant. Our survey of dietary habits revealed that the daily vegetable intake was significantly increased from 157 ± 20 g to 547 ± 17 g and from 183 ± 25 g to 550 ± 22 g in the BST and MST groups, respectively. 

### 3.8. Sensory Evaluation 

Twice during the intervention period, boxes containing MST and BST vegetables were tested by a sensory panel to ensure the vegetables could be distinguished from one another based on their taste. Results from the triangle tests are shown in the supplementary data in Table 6. The assessors evaluated the samples from the MST box as being significantly different compared to samples from the BST box, except for kale tested one time and for pointed cabbage tested one time also.

## 4. Discussion

This randomized, controlled, parallel intervention study was designed to clarify, for the first time, if high dietary intake of selected bitter and strong-tasting *Brassica* and root vegetables could significantly improve key disease characteristics of type 2 diabetic patients compared to the habitual Nordic diet. We found great health improvements in both vegetable groups caused by the daily intake of 500 g root vegetables and cabbages, but interestingly, the bitter and strong-tasting vegetables had the greatest impact on insulin sensitivity, lipid profile, body fat mass, and blood pressure.

Although both vegetables overall improved glycemic control compared to control, the BST vegetables exerted greater beneficial effects on glucose iAUC from the OGTT and fasted glucose compared to the MST and CON groups after 12 months of intervention. Lower levels of plasma glucose during the OGTT indicate an improvement in insulin sensitivity as a result of the high vegetable intake. The BST vegetables exerted a two-fold improvement in insulin sensitivity, as determined by the participant’s response to the glucose load, but this did not achieve significance. In addition, fasting levels of HbA1c were reduced equivalently by the BST and MST vegetable diets. The baseline level of HbA1c in all three groups was above 42.1 mmol/mol, indicating at least an elevated prediabetic level. Studies have demonstrated that weight loss improves glycemic control in diabetic individuals and can lead to remission of diabetes comparable to findings of the present study [25,26,27]. None of the participants changed their medications use during the 3-month clinical trial, excluding medication use as a confounding variable. 

Although the study was not specifically designed as a weight-loss study, both vegetable groups lost a significant amount of weight. It is notable that participants were allowed to eat ad libitum as long as the 500 g of root vegetables and cabbages were included in their daily diet. Weight loss was most likely caused by the root vegetables and cabbages substituting less desirable foods from the participant’s daily diet. Root vegetables and cabbages are rich sources of dietary fibers, and research has shown that food with a high fiber content causes early satiation, enhanced sensation of satiety, decreased subsequent hunger, decrease in insulin response following meals, and slowing of gastric emptying, thus increasing macronutrient absorption [27,28]. The high consumption of vegetables as described above may result in a lower daily food and energy intake, causing the participants to unconsciously lose weight. An intervention causing a weight loss is a principal non-pharmacological method for the prevention and treatment of insulin resistance [29,30]. Positive effects on body weight and overall dietary profile are recognized as a potential way in which F&V can reduce the risk and development of chronic disease [5]. 

The mean baseline BMI in all three groups reveals according to international standards [31] that the participants were obese with an average BMI above 30. DEXA scans facilitated a more in-depth investigation of body composition. DEXA is a precise, accurate, non-invasive, safe, and convenient technique founded on a three-compartment model separating the body into total body mineral mass, fat mass, and lean tissue mass, the latter being the remaining bone-free fat-free tissue mass [32,33]. Both vegetables groups lost approximately the same amount of kilos and cm around the waist after the intervention. 

The clinical conditions commonly associated with T2DM are hypertension and dyslipidaemia, which are critical risk factors for CVD. CVD is the leading cause of morbidity and mortality in individuals affected by T2DM, and the rates of CVD mortality are two to four times higher in diabetic patients compared with the non-diabetic population [26,34,35]. It is highly important for T2DM patients to have BP and lipid levels within the recommended levels [26]. Participants in this study had mean baseline levels of total cholesterol, LDL and HDL cholesterol, and triglycerides that were approximately within the recommended levels. However, different recommendations exist for men and women regarding HDL cholesterol, and both sexes were included as participants. Current guidelines only consider lowering of triglyceride levels to be desirable and not a therapeutic goal [36,37], although a direct association between mean triglyceride levels and long-term total mortality risk in adults with T2DM have been found [38,39]. Lipid profiles were not significantly affected by the intervention. 

The participants’ mean BP at baseline were well adjusted and within the normal range for healthy adults, but approximately 70% of the participants in each group were already on antihypertensive medicine. We used ambulatory BP monitoring with repeated measurements over 24 h, which is less variable and more accurately detects changes in BP than clinical BP measurements. In addition, measurements were conducted in real-life ambulatory conditions, reducing the influence of the “white-coat” effect [40]. The BP-reducing effect in the BST group could be explained by the large weight loss; however, both vegetable groups lost approximately the same amount of weight. If the lowering of the BP in the BST groups was only caused by the loss of body weight, it would be reasonable to expect a similar effect on the BP in the MST group, but this was not the case. The 12 weeks of intervention in BST group lowered BP in ranges comparable with the effect of a DASH [41,42] and a New Nordic Diet [43]. A decline in BP of this magnitude is of great clinical importance, considering that even a small (1–4 mmHg) long-term reduction of BP is estimated to reduce cardiovascular mortality by approximately 5–20% [44,45]. 

From the literature, we know that green leafy vegetables e.g., kale, but also beetroots are among the highest nitrate-accumulating vegetables [46], and it has been postulated that the cardio-protective effects seen from the consumption of green leafy vegetables and beetroots may be due to their high dietary nitrate content, which generates NO independently of the conventional endogenous NO synthase pathway [47,48]. Since hypertension is associated with a diminished endogenous NO production, the increased dietary nitrate may play an important role in the lowering of the BP. The nitrite derived from ingested nitrate provides an intravascular source of NO, which results in vasodilation within the microcirculation, producing a decrease in peripheral resistance [49] and thereby a reduction in BP.

From the NMR spectroscopy, we found that the BST and MST groups were able to lower the levels of lactate in the plasma within the groups. Blood lactate is a measure of the gap between energy expenditure and oxidative capacity. The decline in lactate can be an indicator of the vegetable groups experiencing a better oxidative capacity, hence a better mitochondrial function, and it has previously been found that decreased oxidative capacity is linked to insulin resistance [50]. It was also observed that the BST vegetable diet was capable of altering the amino acid metabolism, as the level of isoleucine was decreased within the group as a result of the vegetable intake. A relation between elevated blood glucose levels and increases in the amino acid isoleucine levels have been demonstrated [51] caused by an abnormal amino acid metabolism due to accumulation of potentially mitotoxic metabolites, leading to mitochondria dysfunction. The lowering of isoleucine levels after the intervention might be caused by an improvement in the complete mitochondrial amino acid metabolism by epigenetic factors affecting gene expression [52].

A panel of biomarkers (notably α- and β-carotene, vitamin C, lutein, zeaxanthin, and β-cryptoxanthin) can be used as indicators of compliance in vegetable intervention trials [53]. We choose β-carotene content as a biomarker of compliance and observed the highest increase in the BST group with a percentage increase of 9.4%, although this was non-significant. Along with self-reported increases in vegetable intake, these methods are good indicator of compliance. However, it has previously been shown that subjects may vary widely in efficiency of carotenoid absorption, including beta-carotene, and that peak plasma response to beta-carotene by the consumption of a large single intake of carrots rich in beta-carotene only produces a small increase in beta-carotene, whereas a single intake of for example broccoli does not change plasma carotenoids, including beta-carotene [54]. These observations may also explain the small changes observed in beta-carotene plasma concentrations in the three intervention groups. Furthermore, the concentration of carotenoids in mild and sweet vegetables and bitter vegetables are considered to be at the same level because the main changes between these vegetables are mainly in the levels of bioactive bitter and strong-tasting phytochemicals such as glucosinolates, polyacetylenes, and phenolic compounds. 

One of the largest public health campaigns and interventions in the Western world in the past decade has been to increase fruit and vegetable intake, and WHO recommends a minimum of 400 g of F&V per day (excluding potatoes and other starchy tubers) for the prevention of chronic diseases [55]. The recommended daily intake of vegetables in Denmark is 300 g based on the Danish Food based Dietary Guidelines [56], but the New Nordic Diet is more specific in its recommendations, stating that a person should eat > 400 g vegetables daily, and of those 400 g vegetables, cabbage should account for >29 g and root vegetables > 150 g, respectively [8]. The advantageous effects of Brassica vegetables on health improvements have been mainly attributed to their content of essential nutrients and health-promoting phytochemicals such as vitamins, minerals, phenolic compounds, phytic acid, and glucosinolates [10], whereas the health-promoting effects of Apiaceae root vegetables such as carrots are most likely attributed to their content of highly bioactive polyacetylenes [19]. The vegetables used in the study were chosen due to their bitter and strong taste, which is likely caused by higher levels of phytochemicals, as many beneficial phytochemicals have a bitter, sour, or astringent taste. Hence, modern consumers, farmers, and breeders tend to select sweeter, more visually attractive, and productive cultivars of crops at the expense of more bitter and healthier ones with higher content of phytochemicals. Davis et al. studied changes in USDA food composition nutritional data from both 1950 and 1999 for 43 different vegetables and fruits, and they found that there may be trade-offs between yield and nutrient content and that cultivars and modern crops are selected based on yield, growth rate, pest resistance, and visual appearance with less or no focus on nutrient content [57].

The type of vegetables has great importance when it comes to dietary management of the risk and development of T2DM. A meta-analysis of prospective cohort studies showed that an increase of 1.15 servings a day of dark-green leafy vegetables was associated with a 14% (HR 0.86, 95% CI 0.77 to 0.97) reduction in risk of T2DM (*p* = 0.01) [6], and the EPIC-InterAct data also indicate an inverse association between root vegetables and diabetes risk (HR 0.87, 95% CI 0.77 to 0.99) [4]. Studies investigating the relationship between vegetable intake and T2DM have reported that intake of green vegetables, rather than total intake of vegetable, is particularly effective at preventing the development of diabetes [6,58]. Another study suggests that vegetable intake of 200 g or more, of which 70 g or more is comprised of green vegetables, correlates with improved HbA1c levels and serum triglycerides levels in elderly patients with T2DM [59]. This highlights the importance of quality as well as quantity when it comes to vegetable intake and health outcomes. Previously, it has been shown that the bitter taste of carrot cultivars is correlated with a high content of phytochemicals such as polyacetylenes [20]; hence, we speculate that the bitter and strong-tasting vegetables have a better quality (higher nutrient/phytochemical content) and therefore a greater health-promoting effect.

Foods are extremely biochemically complex and contain compounds that may interact with one another. Food frequency questionnaires are known to overestimate fruit and vegetable consumption [60]. The whole food hypothesis [14] states that nutrients in isolation may not be sufficient to achieve positive health effects, and whole food should be consumed instead. Root vegetables and cabbages are rich sources of antioxidants such as flavonoids, carotenoids, and other bioactive phytochemicals; ingesting these combined in whole foods may allow for synergistic action, thus providing enhanced health-promoting effects [18,19]. Bioactive phytochemicals such as polyacetylenes or the breakdown products of glucosinolates (isothiocyanates, nitriles, and thiocyanates) are highly bioactive and have previously been shown to exert anti-inflammatory and anti-cancer activity and to stimulate the immune system [20,61,62,63], and in addition, polyacetylenes from Apiaceae root vegetables have been shown to improve glucose uptake in vitro in adipocytes and myotubes [64]. These compounds may also exert their health-promoting effects by stimulating the production of cytoprotective proteins (e.g., phase 2 enzymes), thus activating the endogenous antioxidant defense system, which is also known from other food ingredients [62,65,66]. A possible site where antioxidants can influence health is on mitochondria function, as mitochondria respiration plays a critical role in glucose metabolism. Mitochondria dysfunction has been shown to be associated with metabolic dysregulation, but dietary phytochemicals can optimize mitochondriogenesis and improve oxidative defenses, contributing positively to energy utilization by cells [67]. Phytochemicals can also activate a mild uncoupling of respiration and thereby participate in antioxidant defense; uncoupling proteins are postulated to be the effectors of such defense mechanisms [17]. We have not included the nutritional content of the vegetables in this paper, as we find it beyond the scope of the article, as there are hundreds of compounds for each vegetable, and the vegetables are consumed together with other food components as a part of a normal diet. However, we have analyzed some selected phytochemicals and found a good correlation between bitter/strong taste and amount of e.g., glucosinolates in white cabbage, where the mild/sweet-tasting varieties contained about 7.6 ± 0.8 µmol/g (dry weight) and the bitter/strong testing vegetables contained 13.7 ± 2.6 µmol/g (unpublished data). These data will be published in a separate paper. 

The strengths of the present study include the design: a dietary parallel intervention study with good adherence rate as self-reported by participants and confirmed by biomarkers. A weekly meeting ensured that the research team was in close contact with all the participants. The root vegetables and cabbages were provided to the participants as well as the boxes of foods to the control group. The drop-out rate during the intervention was low in all three groups. All subjects were in a free-living environment and were solely responsible for preparing and cooking the food. The participants were given helpful tools (recipes) so that they would be able to continue the intervention even after the 12 weeks. A high daily intake of vegetables is a cheap way of improving a participant’s lifestyle with no long-term side effects. Lifestyle intervention studies have demonstrated a reduction in the risk of developing T2DM ranging from 30% to 67%, but more importantly, the reduction of diabetes incidence remains after the individual lifestyle counseling was stopped [68].

The vegetables used in the study are easily available and cheap. However, at the present time, it is not possible to buy the seeds of the traditional bitter and strong-tasting cultivars of vegetables. By including the traditional cultivars in the vegetable diets, a broader diversity of taste was represented. This enabled us to create the variation in bitter taste and content of phytochemical, which here was demonstrated to influence T2DM. The vegetables used in the study were chosen solely on their sensory quality, and we do not know the exact content of the nutrients and phytochemicals in the vegetables. Although it is known that polyacetylenes and the breakdown products of glucosinolates are bioavailable [19,69], we have no knowledge about the bioavailability of the different phytochemicals present in the vegetables except for β-carotene. 

A sudden daily intake of 500 g vegetables for 12 weeks is a fairly radical change in diet, so it would be very interesting to see if the same positive results could have been achieved with a lower vegetable intake over a longer intervention period. Available data indicate that a functional food-based diet may be a novel and comprehensive dietary approach for the management of T2DM. Functional foods beyond the basis nutritional functions have potential benefits to promote health and thereby reduce the risk of chronic diseases. Researchers have focused on properties of the bioactive compounds of functional foods in the control of T2DM [70,71]. 

## 5. Conclusions

To conclude, this study clarifies the tremendously positive effect that high daily intake of root vegetables and cabbages has on glucose control and insulin sensitivity in T2DM patients. The results indicated a positive effect on glucose control and beneficial effects on other cardiovascular risk factors, such as BP, body weight, and body composition. Although both vegetable diets were found to improve the health status of participants, the BST exhibited overall greater potential anti-diabetic effect compared to control than MST. The BST diet exerted substantial improvements to key T2D-related parameters compared to MST, i.e., improvements to total body fat (2-fold), total cholesterol (2-fold), fasting glucose (4-fold), and iAUC of glucose. These findings demonstrated significant differences between BST and MST diets for these key T2D parameters. This study clearly exemplifies the beneficial effects of high daily intake of BST vegetables against T2D and its risk factors compared to habitual Nordic diet. BST shows promise as a more effective dietary strategy in the treatment and prevention of T2D compared to MST, although enhanced effects of BST over MST on T2D require further study in larger clinical trials to fully elucidate their preventive potentials. This study may contribute to new dietary recommendations for T2DM patients.

## Figures and Tables

**Table 1:** Cropping and storage strategies to produce vegetables of bitter and strong taste (BST) or mild and sweet taste (MST) sensory quality.

Strategy	Bitter and Strong (BST)	Mild and Sweet (MST)
Traditional cultivars ^1^	Curly kale *Brassica oleracea* L. var. *sabellica* L. ‘Halvhøj Kruset Konserva’ White cabbage *B. oleracea* L. var. *capitata* ‘Dural’ Celeriac *Apium graveolens* var. *rapaceum* ‘Balder Maskot’, ‘Balder Baro’, ‘Balder Bonnet’ Carrot *Daucus carota* var. *sativus* ‘Valoria’	Curly kale ‘Høj Amager Toftø’ White cabbage ‘Amager Høj Grøn Kalida’, ‘Amager Lav Capo’
Modern cultivars ^1^	Carrot ‘Mellow Yellow’ Beetroot *Beta vulgaris* var. *vulgaris* ‘Chioggio’, ‘Burbees’	Celeriac ‘Rowena’ Carrot ‘Bolero’, ‘White satin’ Beetroot ‘Pablo’, ‘Taunus’
Nitrogen and sulfur fertilization ^2^	High (standard) nitrogen and sulfur fertilization of curly kale and pointed cabbage ^3^	Low nitrogen (half) and sulfur (zero) fertilization of curly kale and pointed cabbage ^3^
Storage conditions	Two weeks in ethylene atmosphere of carrot ‘Bolero’	No storage of carrot ‘Bolero’

^1^ At standard fertilization of nitrogen and sulfur according to agronomic practice; ^2^ according to standard agronomic practice; ^3^ modern cultivars ‘Reflex’ and ‘Caraflex’.

**Table 2:** Clinical baseline characteristics of the subjects in the three intervention groups.

		Experimental Diets		
	Control ^a^	Mild and Sweet ^a^	Bitter and Strong ^a^	*p*-Value ^b^
Participants (*n*)	27	28	27	-
Age (years)	63.6 ± 1.3	62.6 ± 1.4	61.6 ± 1.1	0.58
Diagnosis (years)	3.3 ± 0.7	2.8 ± 0.7	3.5 ± 1.3	0.43
Daily vegetable intake (g)	222 ± 23	157 ± 20	183 ± 25	NS
β-carotene (μg/mL plasma) *	0.5 ± 0.0	0.5 ± 0.0	0.5 ± 0.0	NS
Weight (kg)	96 ± 3.8	94 ± 3.3	91.3 ± 3.2	0.75
Waist circumference (cm)	109.2 ± 3.8	104.1 ± 2.6	102.5 ± 2.2	0.26
BMI (kg/m^2^)	33.1 ± 1.3	32.9 ± 1.5	31.8 ± 1	0.73
Total body fat (%)	39.3 ± 1.8	39 ± 2	37.9 ± 1.6	0.85
Plasma glucose (mmol/L)	7.3 ± 0.3	6.7 ± 0.3	7.6 ± 0.3	0.06
HbA_1c_ mmol/mol	48.2 ± 1.0	47.8 ± 1.4	50.4 ± 1.7	0.37
iAUC glucose (mmol/L)	755 ± 59	706.9 ± 62.1	911.1 ± 100.3	0.15
Plasma insulin (pmol/L)	68.8 ± 8.3	67.2 ± 9.6	73.5 ± 7.0	0.87
Plasma glucagon (pg/mL)	64 ± 3.94	64.7 ± 2.96	72.1 ± 3.09	0.17
HOMA-IR	4.1 ± 0.6	3.3 ± 0.5	4.25 ± 0.4	0.39
Plasma cholesterol (mmol/L)	4.4 ± 0.2	4.4 ± 0.2	4.7 ± 0.2	0.58
Plasma LDL–cholesterol (mmol/L)	2.2 ± 0.2	2.4 ± 0.2	2.4 ± 0.2	0.74
Plasma HDL–cholesterol (mmol/L)	1.3 ± 0.1	1.3 ± 0.1	1.3 ± 0.1	0.93
Plasma triglycerides (mmol/L)	2.0 ± 0.3	1.6 ± 0.2	1.6 ± 0.2	0.37
Free fatty acids (mmol/L)	0.5 ± 0.3	0.5 ± 0.3	0.5 ± 0.3	0.56

^a^ Values are presented as mean ± SEM or numbers (%). ^b^ Differences tested between groups at baseline (ANOVA). * *n* = 15 for CON, MST, and BST respectively making a total of *n* = 45.

**Table 3:** Changes (Δ) in fasting insulin, glucagon, and area under the curve (AUC) from the Oral Glucose Tolerance Test regarding insulin (240 min), glucagon (240 min), and HOMA-IR calculations.

			Experimental Diets				
	Control		Mild and Sweet		Bitter and Strong		between Groups
	Changes ^a^	*p*-Value ^b^	Changes ^a^	*p*-Value ^b^	Changes ^a^	*p*-Value ^b^	*p*-Value ^c^
Fasting insulin (pmol/L)	−1.99 ± 4.1	0.63	−7.77 ± 2.8	<0.01	−9.0 ± 3.33	<0.05	0.29
Fasting glucagon (pg/mL)	−2.25 ± 2.19	0.32	−2.23 ± 1.73	0.21	−2.17 ± 1.78	0.28	0.99
AUC insulin_240 min_, (pmol/L ∗ 240 min) ^†^	−632.7 ± 3355	0.85	−2795 ± 2777	0.32	−7199 ± 3476	< 0.05	0.21
AUC glucagon_240 min_ (pg/mL ∗ 240 min)	142.7 ± 561.3	0.80	−159.5 ± 416.2	0.71	−13.6 ± 524.3	0.98	0.92
HOMA-IR ^†^	−0.13 ± 0.42	0.76	−0.46 ± 0.23	0.06	−1.05 ± 0.23	<0.0001	0.057

^a^ Data are presented as mean ± SEM. ^b^ Statistical calculations are within-group differences (paired *t*-test). ^c^ Statistical calculations are between group differences (ANOVA). † denotes use of non-normally distributed data and use of the Kruskal–Wallis test.

**Table 4:** Changes (Δ) in weight, waist circumferences, lean mass, and blood pressure after a 12-week intervention.

			Experimental Diets				
	Control		Mild and Sweet		Bitter and Strong		Between Groups
	Changes ^a^	*p*-Value ^b^	Changes ^a^	*p*-Value ^b^	Changes ^a^	*p*-Value ^b^	*p*-Value ^c^
Weight (kg)	−0.004 ± 0.39	0.99	−2.54± 0.51	<0.0001	−2.4 ± 0.54	<0.0001	<0.01 ^d,e^
Waist circumference (cm)	−3.82 ± 2.29	0.11	−4.00 ± 0.63	<0.0001	−4.45 ± 0.86	<0.0001	0.91
DEXA							
Lean mass (kg)	0.40 ± 0.4	0.25	−0.68 ± 0.3	<0.05	−0.04 ± 0.3	0.90	0.07
24 h Diastolic BP (mmHg)	1.76 ± 1.06	0.11	−1.15 ± 0.93	0.22	−2.12 ± 1.28	<0.05	<0.05 ^f^
24 h Systolic BP (mmHg)	3.16 ± 2.04	0.14	−1.22 ± 1.5	0.42	−3.23 ± 2.23	0.16	0.07

^a^ Data are presented as mean ± SEM. ^b^ Statistical calculations are within-group differences (paired *t*-test). ^c^ Statistical calculations are between group differences (ANOVA). ^d^ control vs. mild and sweet <0.001, ^e^ control vs. bitter and strong <0.01, ^f^ control vs. bitter and strong <0.05.

**Table 5:** Changes (Δ) in lipids, PTH, vitamin D, β-carotene lactate, and isoleucine after a 12-week intervention.

			Experimental Diets				
	Control		Mild and Sweet		Bitter and Strong		between Groups
	Changes ^a^	*p*-Value ^b^	Changes ^a^	*p*-Value ^b^	Changes ^a^	*p*-Value ^b^	*p*-Value ^d^
TC (mmol/L)	−0.03 ± 0.11	0.79	−0.19 ± 0.11	0.11	−0.30 ± 0.12	<0.05	0.26
Trig (mmol/L) ^†^	−0.14 ± 0.11	0.23	−0.11 ± 0.09	0.19	−0.15 ± 0.08	0.07	0.42
LDL (mmol/L) ^†^	−0.01 ± 0.8	0.95	−0.09 ± 0.8	0.24	−0.14 ± 0.8	0.08	0.23
FFA (mmol/L)	0.05 ± 0.04	0.10	0.04 ± 0.02	0.30	−0.05 ± 0.16	0.13	0.07
HDL (mmol/L)	−0.01 ± 0.03	0.74	−0.04 ± 0.04	0.28	−0.01 ± 0.5	0.86	0.8
PTH (mmol/l)	−0.37 ± 0.24	0.14	0.05 ± 0.2	−0.56	0.02 ± 0.2	0.45	0.23
Vitamin D2 + D3 (ng/mL)	−11.18 ± 4.1	0.11	−13.96 ± 2.26	<0.0001	−17.04 ± 3.74	<0.0001	0.49
β-carotene (%) ^c^	1.26 ± 3.4	NS	2.58± 3.6	NS	9.44 ± 5.1	NS	NS
Lactate (%)	−7.7 ± 4.3	NS	−8.8 ± 5.3	<0.05	−13.6	<0.01	NS
Isoleucine (%)	−1.3 ± 5.9	NS	0.74 ± 5.8	NS	−11.6 ± 4.5	<0.05	

^a^ Data are presented as mean ± SEM. ^b^ Statistical calculations are within-group differences (paired *t*-test). ^c^ *n* = 15 in each group. ^d^ Statistical calculations are between group differences (ANOVA). † denotes use of Kruskal–Wallis test.

**Table 6:** Sensory evaluation of the vegetables used in the BST and MST groups by a professional sensory panel using a triangle test for each vegetable type.

		Year 1		Year 2	
Date		27.9	15.11	25.09	13.11
Vegetable	Groups	*p*-Value	*p*-Value	*p*-Value	*p*-Value
Carrot	Sweet vs. bitter	<0.0001	<0.0001	<0.001	<0.0001
Celeriac	Sweet vs. bitter	<0.0001	<0.0001	<0.0001	<0.0001
Kale	Sweet vs. bitter	<0.0001	<0.001	<0.0001	NS
Pointed cabbage	Sweet vs. bitter	<0.0001	NS	0.0015	<0.01
White cabbage	Sweet vs. bitter	NI ^a^	<0.0001	NI ^a^	<0.05

Determination of sweet vs. bitter sensory characteristics of vegetable cultivars used in the study by a professional sensory panel. Significant *p* value denotes a significant difference between the panels’ assessment of the bitter and sweet characteristics of the respective paired sweet and bitter cultivars. ^a^ NI = Not included.

## Data Availability

The data presented in this study are available on request from the corresponding author. The data are not publicly available due to GDPR and data protection.
